# Antifungal Activity in Compounds from the Australian Desert Plant *Eremophila alternifolia* with Potency Against *Cryptococcus spp.*

**DOI:** 10.3390/antibiotics8020034

**Published:** 2019-03-31

**Authors:** Mohammed A. Hossain, Israt J. Biva, Sarah E. Kidd, Jason D. Whittle, Hans J. Griesser, Bryan R. Coad

**Affiliations:** 1 Future Industries Institute, University of South Australia, Mawson Lakes, South Australia 5095, Australia; mohammed.hossain@mymail.unisa.edu.au (M.A.H.); israt.biva@mymail.unisa.edu.au (I.J.B.); Hans.Griesser@unisa.edu.au (H.J.G.); 2 National Mycology Reference Centre, SA Pathology, Adelaide, South Australia 5000, Australia; Sarah.Kidd@sa.gov.au; 3 School of Engineering, University of South Australia, Mawson Lakes, South Australia 5095, Australia; Jason.Whittle@unisa.edu.au; 4 School of Agriculture, Food and Wine, University of Adelaide, Adelaide, South Australia 5064, Australia

**Keywords:** Diterpenoids, antifungal, wound healing, serrulatane, fungi, *Eremophila*, *Cryptococcus*, disk diffusion, broth microdilution

## Abstract

Plant metabolites that have shown activity against bacteria and/or environmental fungi represent valuable leads for the identification and development of novel drugs against clinically important human pathogenic fungi. Plants from the genus *Eremophila* were highly valued in traditional Australian Aboriginal medicinal practices, and *E. alternifolia* was the most prized among them. As antibacterial activity of extracts from *E. alternifolia* has been documented, this study addresses the question whether there is also activity against infectious fungal human pathogens. Compounds from leaf-extracts were purified and identified by 1- and 2-D NMR. These were then tested by disk diffusion and broth microdilution assays against ten clinically and environmentally relevant yeast and mould species. The most potent activity was observed with the diterpene compound, 8,19-dihydroxyserrulat-14-ene against *Cryptococcus gattii* and *Cryptococcus neoformans*, with minimum inhibition concentrations (MIC) comparable to those of Amphotericin B. This compound also exhibited activity against six *Candida* species. Combined with previous studies showing an antibacterial effect, this finding could explain a broad antimicrobial effect from *Eremophila* extracts in their traditional medicinal usage. The discovery of potent antifungal compounds from *Eremophila* extracts is a promising development in the search for desperately needed antifungal compounds particularly for *Cryptococcus* infections.

## 1. Introduction

Plants are an abundant source of secondary metabolites, some of which possess in vitro antimicrobial activity [1,2]. Australian aboriginal peoples are known to have used at least around 70 plant species as medicines. In the more arid regions of Australia, various species of the plant genus *Eremophila* (Scrophulariaceae) formed the basis for the most important traditional medicines used for treatment of wounds, sore throats, microbial infections, fever, painful ears, scabies and other skin related infections [3,4,5]. This large genus of Australian plants, with more than 217 species [6,7], has been of interest for phytochemical investigations, and has a wide variety of classes of secondary metabolites [8]. These include terpenes, flavonoids, fatty acids, sterols and lignans, including prevalent diterpenoids, among which the serrulatane skeleton is common [4].

Previously, a number of serrulatane diterpenes were identified as antibacterial compounds [9,10,11,12,13,14,15]; including from *Eremophila alternifolia* R.Br, which figures prominently among traditional Australian aboriginal medicinal plants and is one of the most active species that has been tested and reported [15]. This previous study showed that this highly resinous species possessed interesting antibacterial activity against Gram-positive bacteria including multidrug-resistant clinical isolates of *Staphylococcus aureus*. However, given that the causes of infectious diseases amongst traditional and remote aboriginal users is generally not known, it is worth investigating the possible contribution of human fungal pathogens to disease, particularly because there has been limited recognition of the importance of fungal diseases despite causing immense human suffering [16]. Hand-in-hand with this idea is the observation that many species of *Eremophila* are highly resistant to fungal attack by environmental moulds [17]. Thus, an investigation of the antifungal properties of these compounds and their putative role in treatment of diseases is a worthy topic, and timely given the human impact of fungal diseases and the desperate need to develop new antifungals drugs [18].

The incidence of invasive fungal infections has markedly increased in the last 30–40 years and is recognised as a global healthcare crisis [19]. Data from the United States show that the rates of life-threatening fungal infections increased by more than 200 % between 1979 and 2000 [20]. Amongst the yeasts, more than 20 species have been implicated in invasive candidiasis or candidaemia, with *Candida albicans* being the most common. There has also been increasing awareness of cryptococcal diseases in immunocompromised patients [21]. For example, cryptococcosis is the second-most common AIDS related infection in sub-Saharan Africa, causing approximately 6 million deaths annually [22]. With moulds, the lack of diagnostic tools for early screening of *Aspergillus*-related illness, contributes to its status as a life-threatening disease [23].

Natural products are a key source for novel antimicrobials but the majority of drug discovery has been focussed on antibacterial agents and antivirals; in contrast, relatively few antifungal agents were obtained from natural sources. In 33 years, only four new antifungal compounds were discovered from natural sources, representing approximately 4 % of all such naturally-derived antimicrobials, well behind the discovery of new antibacterial and antiviral agents [24]. Particularly against the spectre of rising drug resistance, the discovery of new antifungals from natural sources is desperately needed, especially if they inhibit fungi-specific pathways or act on novel cellular targets [25,26].

The present study was undertaken to extend previous investigations into the antimicrobial activity of *Eremophila* species, now using plant extracts and chemical synthesis to examine potency against human *fungal* pathogens. We discuss the screening of antifungal drugs using two testing methods: the disk diffusion and broth microdilution methods. Importantly, we show how the potent antifungal activity against *Cryptococcus* spp. could have been missed if too much reliance was placed on the disk diffusion method as an exclusive initial assessment of activity.

## 2. Results

### 2.1. Identification of Compounds

**Figure 1:**
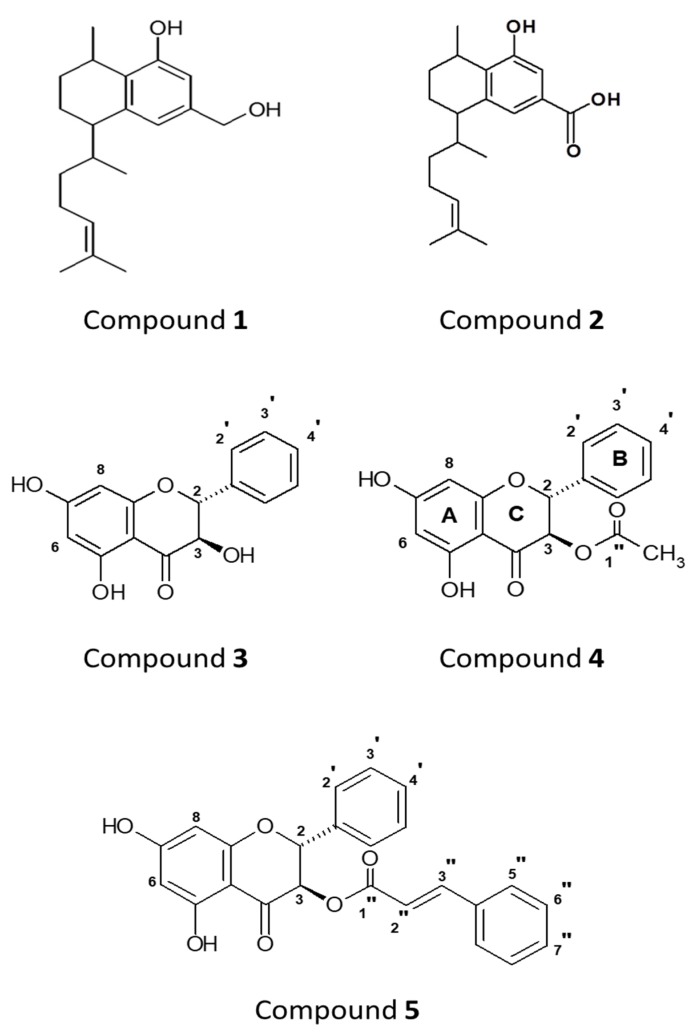
Chemical structures. Compound 1: 8, 19-dihydroxyserrulat-14-ene. Compound 2: 8-hydroxyserrulat-14-en-19-oic acid. Compound 3: Pinobanksin. Compound 4: Pinobanksin-3-acetate. Compound 5: Pinobanksin-3-cinnamate.

Extraction and purification of plant extracts led to five compounds of interest labeled 1–5 (Figure 1).

**Figure 2:**
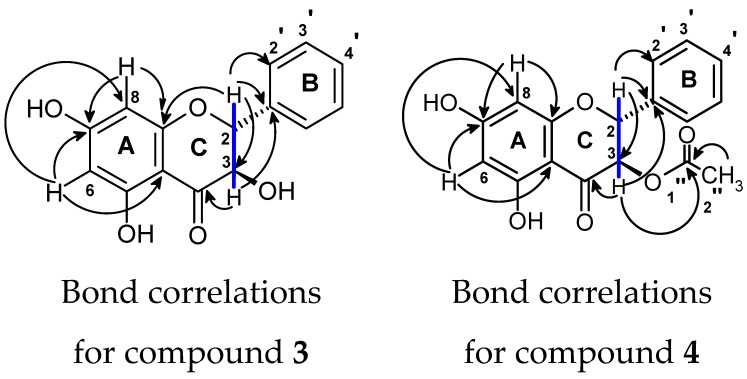
Key heteronuclear multiple bond correlations (→) and homonuclear correlations (―) in compounds 3 and 4 interpreted from 2-D NMR.

The structures of these compounds were identified by analysing 1D and 2D NMR data and comparing with a previously published report [15]. NMR spectra for extracted compounds 1, 2, and 5 were straightforward and confirmed their identification. Figure 2 shows the assigned heteronuclear bond correlations that resulted in identification of compounds 3 and 4. Compound 2 was reduced to compound 1 and this was confirmed by NMR and agreed with published data [27]. 

### 2.2. Antifungal Screening Using the Disk Diffusion Assay

Disk diffusion was used as a preliminary screening technique for antifungal activity. The zones of inhibition against three fungal strains are presented in Table 1 for compounds along with those for the standard drugs caspofungin, nystatin and amphotericin B. Compounds 1–5 showed a moderate zone of inhibition of 7–8 mm against *C. albicans*, 11–13 mm against *C. gattii*, and 10–12 mm against *C. neoformans*. No zone of inhibition could be discerned for either compound against the other seven fungal isolates. 

### 2.3. Antifungal Susceptibility using the Broth Microdilution Assay

The minimum inhibitory concentrations (MIC) for yeasts and minimum effective concentrations (MEC) for moulds obtained from broth microdilution assays with various fungal pathogens are summarised in Table 2. Compound 1 exhibited strong activity against standard strains of *C. gattii* and *C. neoformans* (4 µg/mL)*,* which is comparable to that of the reference polyene compounds nystatin and amphotericin B, and less than that of the reference compound caspofungin. Compound 1 also exhibited activity against *C. albicans* and *C. krusei* (4–8 µg/mL), and against *C. parapsilosis* (8–16 µg/mL), *C. tropicalis* (16 µg/mL), *C. lusitaniae* (16–32 µg/mL) and *C. glabrata* (16–32 µg/mL). Compound 5 was less potent than Compound 1 by 2 to 8 times, depending on the fungal species tested. Compounds 2–4 were much less active (128–256 µg/mL) in vitro. All compounds exhibited little activity against the moulds *A. niger* and *A. fumigatus* (64–˃512 µg/mL).

## 3. Discussion

Over the past three decades there has been an increase in the incidence of invasive fungal infections particularly in immunocompromised human patients, including those caused by fungi with acquired and intrinsic antifungal resistance [19]. For superficial infections, dermatomycoses remains a prevalent and widespread health concern [28]. A critical need for new and improved antifungal agents was the motivation to investigate compounds from *Eremophila spp.* for antifungal activity against human fungal pathogens.

The comparison of activity of our compounds with standard antifungal drugs can best be discussed using the results from the broth microdilution assay. Here, compound 1 had very good in vitro activity particularly against *C. neoformans* and *C. gattii* species, and moderate activity against *Candida* species (*C. albicans*, *C. krusei* and *C. glabrata*). Notably, with *Cryptococcus* sp., the modal MIC of 4 µg/mL was lower than that of both caspofungin and nystatin, and just higher than that for amphotericin B. This promising activity and the marked differences of activity against the different fungal genera warrant further investigation to study in detail the molecular mechanism of inhibition against medically relevant yeasts.

While we have not conducted a detailed mechanistic study here, a comparison of data between compounds and related studies pertaining to bacteria forms a basis for future investigations. When comparing the activity of the structurally similar compounds 1 and 2 (broth microdilution data, Table 2) the alcohol substituent appears to greatly increase activity when compared to the carboxylic acid substituent in the same position. Similar results were found in previous work where these two compounds were tested against various Gram-positive bacteria [10,29]. The mechanism of action of compound 2 was studied in detail with Gram-positive bacteria and it was shown to inhibit the macromolecular biosynthesis pathways and compromise cell membrane integrity [30]. Interestingly, compound 2 did not have activity against Gram-negative bacteria, which might suggest a crucial role of activity against the cell wall membrane in bacteria.

A recent study showed that monoterpenoids exert the antifungal effect by destabilizing the cell membrane [28]. Although the mechanisms responsible for antifungal activity of diterpenoids have not yet been elucidated, it could be hypothesised that these diterpenoids (1 and 2) might act similarly to monoterpenoids. Therefore, semi-synthetic derivatisation of the structural backbone of the compounds studied here may provide further antifungal lead compounds and provide insights into their mechanisms of action. 

Another aspect of this investigation compares two procedures for testing antifungal activity. Disk diffusion and broth micro dilution assays both have advantages and disadvantages in terms of cost, labour, ease of use and interpretation. Often, the disk diffusion method is used as a primary screen for microbial activity, with secondary screening (e.g., broth microdilution) being conducted after an indicative response. In our evaluation, we carried out both methods and compared the results. Interestingly, we found disparate results between these two methods, particularly notably so for compound 1 against the yeasts. For *Candida* species, standard drugs resulted in relatively wide zones of inhibition exceeding ~18 mm in every case, as expected, but for compound 1, no inhibition zone was observed for any species (except *C. albicans*, ca. 8 mm). An initial screen using this method might have concluded very poor *Candida* inhibition compared to standard drugs. However, the broth microdilution assay showed that Compound 1 did indeed have some activity versus all *Candida* species. Particularly notable is the modal MIC of 1 against *C. krusei* (8 µg/mL) which is the same order of magnitude as for amphotericin B (1 µg/mL), but the compound did not show a visible zone of inhibition. Also interesting was the much smaller zone of inhibition of 1 against *Cryptococcus* spp. compared to standard antifungal drugs, which could be interpreted as relatively poor activity; however, by broth microdilution, the quantified MIC was comparable to many of the reference drugs. 

Such disparity between broth microdilution and disk diffusion assays could be explained by the mobility of the compounds through the semi-solid agar matrix. Diffusional mobility is influenced by the size and polarity of the molecule. This interpretation is supported by a previous study showing that the lower polarity of natural compounds affected the diffusion of compounds onto the culture medium [31,32,33,34]. These results show the importance of using multiple assay methods to investigate screening of new compounds.

This report provides evidence of antifungal activity from extracts of *Eremophila alternifolia* and describes compound isolation and purification. Beyond the scope of this initial report, we suggest future studies to more thoroughly investigate the utility of using these compounds to treat clinical infections. For example, it would be worthwhile to explore possible drug synergism with drugs currently used for fungal infections [25,35,36], and there is some evidence to suggest that terpenes may have use in combination therapy against dermatophytes and yeasts [28]. In this experiment, we screened extracted compounds against typed strains; however, future work should also investigate whether the active compounds could treat drug-resistant clinical isolates. Novel agents that have use in treating clinically recalcitrant infections would be highly valued.

Finally, with any new antimicrobial compound, it would also be valuable to investigate the possibility that fungal pathogens could develop resistance. In our study, we did not observe early indicators of such resistance (such as the appearance of resistant colonies within the zone of diffusion). However, a more thorough future investigation should look at repeated culturing of pathogens in the presence of sub lethal concentrations to see whether resistant phenotypes might be generated.

## 4. Materials and Methods

### 4.1. Collection and Description of Plant Materials

*Eremophila alternifolia* grows in a variety of habitats over a wide range of central Australia and arid zones of Western Australia and South Australia. Plants have alternate leaves and tubular flowers with a variety of colours including purple, red, pink, white, cream or yellow, usually appearing in early winter to early autumn. Other names include poverty bush, narrow-leaf fuchsia bush and native honeysuckle [6,37,38]. According to The Plant List website (www.theplantlist.org) *E. alternifolia* R.Br. is an unresolved name (synonymous with *Bondtia alternifolia* Kuntze and *E. alternifolia* var. latifolia Benth).

Leaves of *E. alternifolia* were collected in September 2013 from plants grown in cultivation on a private property near Dutton, South Australia. The morphology of the cultivated plants was identical to that of wild plants occurring in the area and further north in South Australia. A voucher specimen (AD 271534) was deposited at the State Herbarium of South Australia, Adelaide where species identity was confirmed Dr. R.J. Chinnock, *Eremophila* taxonomist.

### 4.2. Solvents and Reagents

All solvents were analytical, spectroscopic or HPLC grade such as acetone analytical reagent (AR) grade, methanol AR grade, methanol HPLC grade, dichloromethane AR grade and hexane AR grade, formic acid, sulphuric acid reagent grade, glacial acetic acid, p-anisaldehyde, dimethyl sulfoxide (DMSO) and obtained from Merck (Darmstadt, Germany and Australia). Silica-gel 60 F254 aluminium plates (Merck, Germany), were used for thin layer chromatography (TLC) to detect compounds of interest. For visualisation of separated compounds on TLC-plates, anisaldehyde spray reagent was freshly prepared (0.5% *v/v* p-anisaldehyde in 5% *v/v* sulphuric acid, 5% *v/v* glacial acetic acid in ethanol) and used.

### 4.3. Equipment/Instruments Used

A rotary evaporator, R-200 (Büchi, Flawil, Switzerland), a vacuum pump V-700 (Büchi), a vacuum controller V-850 (Büchi), and a heating bath B-491 (Büchi) were used for evaporating solvents from crude extract and subsequent fractions. Various chromatographic techniques were used for separating fractions and isolating compounds, for example, a Sephadex LH-20 (Sigma, St. Louis, MO, USA), 270 × 45 mm glass chromatographic column, silica gel (60 Å pore size, Merck, Germany), and rotating disc chromatography (RDC) (1 mm). Further separations and isolation of compounds were performed by HPLC, consisting of a two-pumps LC-8A unit (Shimadzu, Kyoto, Japan), by using Activon Gold pack normal phase (NP)-semi-preparative (25 × 1 cm, silica) HPLC columns on a Shimadzu LC-6A system and reverse phase (RP) analytical HPLC column (250 × 4.60 mm, 3 µ, C18) on Shimadzu UFLC system with a UV/VIS detector SPD-20A (Shimadzu), a communication bus module CBM-20A (Shimadzu), fraction collector FRC-10A (Shimadzu), software LC Solution (Shimadzu). For identifying the structures of compounds, NMR data (both one- and two- dimensional spectra) were obtained on a Brüker Avance III 500 MHz spectrometer. 

### 4.4. Extraction and Isolation 

Fresh resinous leaves (1.1 kg) of *E. alternifolia* were extracted in acetone overnight in a closed vessel (1 L). The solvent was decanted and evaporated *in vacuo* to dryness (40 °C) to give extract (27 g), which was washed with n-hexane:EtOAc (50:50) to obtain a filtrate (20 g). This filtrate was dissolved in 200 mL MeOH:H_2_O (7:3), exhaustively extracted with *v/v* 5% and 25% CH_2_Cl_2_ in hexane and then with 100% CH_2_Cl_2_ to yield the 5% CH_2_Cl_2_ fraction, the 25% CH_2_Cl_2_ fraction and the 100% CH_2_Cl_2_ fraction, respectively.

**Figure 3:**
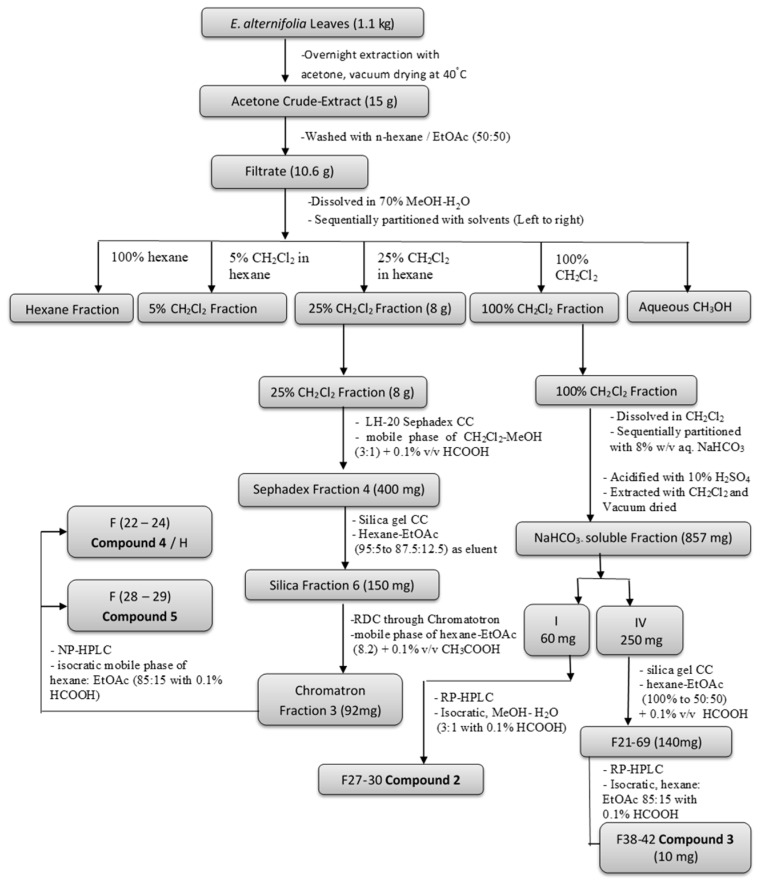
Flow diagram of the compound isolation process from the crude extract of *E. alternifolia.* The structures of the numbered compounds are given in Figure 1.

A portion of crude leaf-extract (5 g) was re-dissolved in MeOH:H_2_O (7:3) and further partitioned sequentially first with comparatively weaker base, an 8% NaHCO_3_ solution (2 times, 100 mL each), and then with stronger base, a 5% NaOH solution (2 times, 100 mL each). This led to three portions, viz., the 8% NaHCO_3_ portion, the 5% NaOH portion, and the CH_2_Cl_2_/hexane portion, respectively. After acidification with conc. H_2_SO_4_, the basic portions 8% NaHCO_3_ and 5% NaOH were extracted with CH_2_Cl_2_ (3 times, 100 mL each) to yield the NaHCO_3_-soluble, the NaOH-soluble, and the neutral CH_2_Cl_2_ fractions. All fractions were dried under vacuum. The compound isolation followed the procedure that is detailed in our previous article [15]. The major partitioned fractions, the 25% CH_2_Cl_2_ fraction and the NaHCO_3_-soluble fraction, were preliminarily subjected to various column chromatographic (CC) separation techniques, such as Sephadex, silica gel CC and RDC, resulting in simplified fractions, and further separated through HPLC by using different columns in an RP / NP system for isolating compounds from *E. alternifolia*. A summary flow diagram of the isolation of compounds is given in Figure 3 and structures of the compounds are given in Figure 1. Additionally, a greater amount of compound 2 (8-hydroxyserrulat-14-en-19-oic acid) was extracted from the 25% CH_2_Cl_2_ fraction for further synthesis work. The pooled silica fraction SF1-SF2 was further separated through RP-HPLC using an isocratic mobile phase of MeOH:H_2_O (3:1 with 0.1% HCOOH), flow rate 2 mL/min, collecting 35 × 2 mL, where fractions 24–31 gave compound 2 (12 mg) as a white powder.

### 4.5. Chemical Synthesis

**Figure 4:**
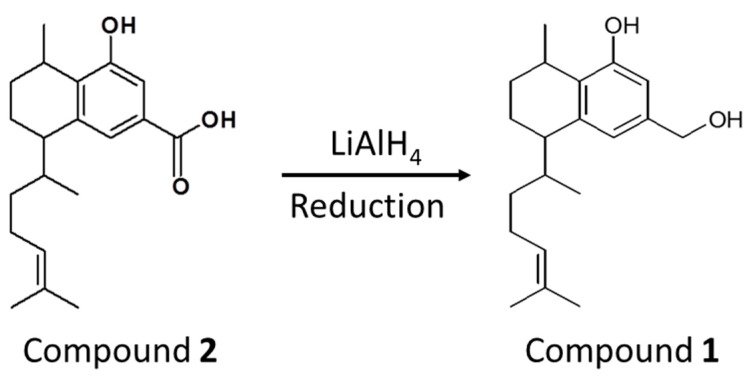
Reduction of compound 2 to compound 1.

The hydroxyl serrulatane, Compound 1, was difficult to extract in high purity even with the use of HPLC, while Compound 2 was both more abundant and easier to isolate. Hence, reduction using a tenfold excess of lithium aluminium hydride (LiAlH_4_) was used to convert Compound 2 into Compound 1 as shown in Figure 4 [39]. This reaction proceeded without the need for protection of the phenolic hydroxyl group.

### 4.6. Fungal Strains

Isolates of ten clinically and environmentally relevant fungal species, *Candida albicans* (ATCC 90028), *Candida tropicalis* (ATCC 750), *Candida parapsilosis* (ATCC 22019), *Candida glabrata* (ATCC 90030), *Candida krusei* (ATCC 6258), *Candida lusitaniae* (ATCC 42720), *Cryptococcus gattii* (ATCC 32609), *Cryptococcus neoformans* (ATCC 90113), *Aspergillus fumigatus* (ATCC MYA 3626) and *Aspergillus niger* (NMRC 14-41711737) were used. All isolates were stored as glycerol stocks at −80 °C until required, and then grown on Sabouraud’s dextrose agar with antibiotics. 

### 4.7. Antifungal Susceptibility Assays

Standard broth microdilution and disk diffusion methods were followed in accordance with the Clinical & Laboratory Standards Institute documents (CLSI M27-A3 and M38-A2 for broth microdilution methods for yeasts and moulds respectively; and M44-A2 and M51-A for disk diffusion method for yeast and moulds respectively) for determining antifungal activity [40,41,42,43]. Clinical antifungal drugs caspofungin (Selleckchem.com, USA), amphotericin B (Sigma-Aldrich, USA) and nystatin (TOKU-E, USA) were used as reference controls. These standard drugs were stored at −20 °C. Concentrated stock solutions (10 mg/mL) were made by first dissolving compounds in 0.1 mL in DMSO and then diluting to 1 mg/mL by adding 0.9 mL 0.85% saline.

In vitro antifungal activities of pure compounds 1–5 were evaluated against the eight yeast and the two mould species. Antifungal susceptibility was assessed by determining zones of inhibition as well as the MIC.

#### 4.7.1. Zone of Inhibition Assays

Single colonies of *C. albicans, C. tropicalis, C. parapsilosis, C. glabrata, C. krusei, C. lusitaniae, C. neoformans* and *C. gattii* were each suspended in sterile 0.85% saline and adjusted to 0.5 McFarland while conidiophores of *A. fumigatus* and *A. niger* were suspended in 3 mL of sterile 0.85% saline and adjusted to optical density 0.09–0.13 at 530 nm. For disk diffusion assays, blank Whatman paper (6 mm) antibiotic assay disks were impregnated with 12 µg of the test and reference compounds and allowed to dry at room temperature. A sterile cotton swab was dipped into each adjusted fungal suspension and used to cover uniformly the surface of a Mueller-Hinton with glucose and methylene blue (MHGMB) agar plate. Antifungal disks were placed on the inoculated MHGMB plates used. Control disks without antifungal drugs or sample compounds were also placed on the plate. The plates were incubated for 24 h at 35 °C before measuring the diameter of the zone of inhibition in millimetres. 

#### 4.7.2. Broth Microdilution Assays

For broth microdilution assays 96 well (U-shaped) microdilution trays were set up with serial, 2-fold dilutions of each test compound, as well as the three reference antifungal agents. The final concentrations of test compounds and standard drugs ranged from 0.001–512 µg/mL and 0.03–16 µg/mL respectively. Drug-free growth control and sterility control wells were incorporated into each tray. Plates were inoculated, yielding final test concentrations colony forming units (CFU) of 0.5 × 10^3^ –2.5 × 10^3^ CFU/mL (yeasts) or 0.4 × 10^4^–5 × 10^4^ CFU/mL (moulds) and incubated for 24–48 h at 35 °C. MIC endpoints were determined visually as the lowest drug concentration with ˃50% reduction (yeasts) or absence (moulds) of fungal growth as per CLSI guidelines [40,41]. In the case of caspofungin activity against moulds, endpoints were determined as minimum effective concentrations (MEC), by the formation of aberrant growth, i.e., compact hyphal balls, as per CLSI M38-A2 specifications [41]. Experiments were performed in triplicate.

## 5. Conclusions

Compounds extracted and semi-synthesised from the Australian plant species *E. alternifolia* exhibited moderate to potent antifungal activities against clinically important fungal pathogens. Notably, the observed anticryptococcal activity of compound 1 was comparable to amphotericin B in vitro. This compound therefore may represent a new lead for a drug development process to treat infections caused by *Cryptococcus* and other yeast species, either alone or in combination with established therapies. Interestingly, in contrast to the broth microdilution assay, the disk diffusion assay was a poorly predictive initial screen for these compounds. Further study is needed to investigate the mechanism of action, pharmacokinetics, and cytotoxicity of these compounds in the context of treating fungal diseases.

## Figures and Tables

**Table 1:** Antifungal activity of compounds 1–5 against a range of fungal species as determined by the disk diffusion method.

Fungal Strains	Incubation Time (hrs)	Zone of Inhibition* (mm)							
		Compounds					Standard Drugs		
		1	2	3	4	5	CAS	NYS	AMB
Yeasts									
*Candida albicans* (ATCC 90028)	24	7.7 ± 0.6	7.3 ± 0.6	7.1 ± 0.3	7.3 ± 0.3	7.3 ± 0.3	22.3 ± 0.6	26.5 ± 0.5	20.7 ± 0.6
*Candida tropicalis* (ATCC 750)	24	NZ	NZ	NZ	NZ	NZ	30.0 ± 1.7	25.7 ± 0.6	18.0 ± 1.0
*Candida parapsilosis* (ATCC 22019)	24	NZ	NZ	NZ	NZ	NZ	25.3 ± 1.2	17.3 ± 0.6	20.7 ± 0.6
*Candida glabrata* (ATCC 90030)	24	NZ	NZ	NZ	NZ	NZ	26.3 ± 0.6	25.6 ± 0.6	17.6 ± 0.6
*Candida krusei* (ATCC 6258)	24	NZ	NZ	NZ	NZ	NZ	25.6 ± 0.6	21.0 ± 1.0	18.3 ± 0.6
*Candida lusitaniae* (ATCC 42720)	24	NZ	NZ	NZ	NZ	NZ	24.0 ± 1.0	24.5 ± 1.3	21.5 ± 0.5
*Cryptococcus gattii* (ATCC 32609)	24	12.7 ± 0.6	11.6 ± 0.6	11.1 ± 0.3	11.3 ± 0.6	12.5 ± 0.5	14.0 ± 0.5	28.3 ± 0.6	29.0 ± 1.7
*Cryptococcus neoformans* (ATCC 90113)	24	11.1 ± 0.6	10.0 ± 1.0	10.8 ± 0.3	11.8 ± 0.3	11.7 ± 0.6	13.3 ± 0.6	24.0 ± 1.7	20.3 ± 1.2
Moulds									
*Aspergillus fumigatus* (ATCC MYA 3626)	24	NZ	NZ	NZ	NZ	NZ	31.7 ± 2.5	17.3 ± 1.1	20.0 ± 1.7
*Aspergillus niger* (NMRC 14-41711737)	24	NZ	NZ	NZ	NZ	NZ	26.3 ± 1.2	22.3 ± 1.2	22.7 ± 1.5

Abbreviations: CAS, caspofungin; NYS, nystatin; AMB, amphotericin B; NZ, no zone. * Values are mean inhibition zone diameter (mm) ± standard deviation of three replicates.

**Table 2:** Antifungal activity of compounds 1–5 against a range of fungal species as determined by the broth microdilution method.

Fungal Strains	Incubation Time (hrs)	MIC (µg/mL) (Modal MIC) *							
		Compounds					Standard Drugs		
		1	2	3	4	5	CAS	NYS	AMB
Yeasts									
*Candida albicans* (ATCC 90028)	24	4–8 (4)	128–256 (256)	128–256 (256)	64–128 (128)	8–16 (16)	0.06–0.12 (0.06)	1–4	0.5–2
*Candida tropicalis* (ATCC 750)	24	16	64–128 (64)	128–256 (256)	64–128 (128)	16–32 (32)	0.06–0.12 (0.12)	2–4 (2)	0.5–1 (0.5)
*Candida parapsilosis* (ATCC 22019)	48	8–16 (16)	256	128–512	128–256 (256)	64–128 (128)	0.25–0.5 (0.5)	2–4 (4)	1–2
*Candida glabrata* (ATCC 90030)	48	16–32 (16)	128–256 (256)	256–512 (512)	256–512 (256)	32–64 (64)	0.25–0.5 (0.25)	1–2 (2)	0.5–1 (0.5)
*Candida krusei* (ATCC 6258)	48	4–8 (8)	256	256–512 (512)	128–256 (256)	16–32 (32)	2–4 (2)	0.5–2	1–2 (1)
*Candida lusitaniae* (ATCC 42720)	48	16–32 (16)	128–256 (256)	128–512	64–128 (128)	64–128 (64)	0.5–1 (0.5)	2–4 (4)	2–4 (2)
*Cryptococcus gattii* (ATCC 32609)	48	4	128–256 (256)	256–512 (512)	128–256 (128)	16–32 (32)	8	2–4 (4)	1–2 (1)
*Cryptococcus neoformans* (ATCC 90113)	48	4	256	256–512 (512)	128–256 (256)	32–64 (64)	8	2–8	0.5–2 (0.5)
Moulds									
*Aspergillus fumigatus* (ATCC MYA 3626)	48	˃ 512	˃ 512	˃ 512	˃ 512	˃ 512	0.03–0.06 (0.03)	4–8 (4)	1–4 (1)
*Aspergillus niger* (NMRC 14-41711737)	48	64	128	˃ 512	˃ 512	˃ 512	0.12 (0.06–0.25)	4–8 (4)	0.12–1

Abbreviations: CAS, caspofungin; NYS, nystatin; AMB, amphotericin B; * Range and (mode) of MICs obtained from triplicate experiments. Caspofungin endpoints determined as MIC for yeasts and MEC for moulds.
